# Exploring the nonlinear relationship between body mass index and health-related quality of life among adults: a cross-sectional study in Shaanxi Province, China

**DOI:** 10.1186/s12955-015-0347-9

**Published:** 2015-09-23

**Authors:** Yongjian Xu, Zhongliang Zhou, Yanli Li, Jinjuan Yang, Xiaoyuan Guo, Jianmin Gao, Ju’e Yan, Gang Chen

**Affiliations:** School of Public Health, Health Science Center, Xi’an Jiaotong University, P.O Box 86, No. 76 West Yanta Road, Xi’an, Shaanxi 710061 China; School of Public Policy and Administration, Xi’an Jiaotong University, Xi’an, China; School of Medicine, Flinders University, Adelaide, Australia

**Keywords:** Health-related quality of life, Body mass index, EQ-5D, Underweight, Obese

## Abstract

**Introduction:**

China is a country facing the “double burden” of both obesity and underweight. The objective of this study was to explore the relationship between body mass index (BMI) and health-related quality of life (HRQOL) in adults from Shaanxi Province.

**Methods:**

The data were derived from the fifth Health Service Survey of Shaanxi Province, which was part of China’s National Health Service Survey (NHSS), conducted in 2013. The HRQOL was assessed using the three-level EQ-5D questionnaire and scored based on a recently developed Chinese-specific tariff. Semiparametric regression models were adopted to explore the non-linear relationship between continuous BMI and overall HRQOL scores. Logistic regression models were further undertaken to assess the relationship between categorized BMI and five dimensions of HRQOL.

**Results:**

Among the study sample (*n* = 37,902), 77 % of men and 75 % of women were assigned to normal weight, according to the WHO International classification. There were statistical significant nonlinear relationships between BMI and HRQOL, with optimal HRQOL achieved at a BMI of near 23 kg/m^2^ for men and 24 kg/m^2^ for women. Before BMI reached optimal HRQOL, the EQ-5D utility scores were increasing faster among men than the women, whilst after the BMI value reached the optimal utility scores, women showed a faster decline in utility scores than men. With adjustments for socio-demographic, physical activity and co-morbidities, obese respondents were more likely to suffer from physical rather than mental problems. Underweight respondents were significantly more likely to report having any problems in all five dimensions of the EQ-5D, whilst the magnitudes of odds ratios were consistently larger for men than women.

**Conclusion:**

There was an inverse U-shaped association between continuous BMI and overall HRQOL scores, meaning that both underweight and obesity were associated with lower HRQOL. The relationship between BMI and HRQOL varied between sexes. Underweight respondents had a higher risk of suffering from both physical and mental problems. Interventions aimed to tackle the prevalence of underweight should be put into action in Shaanxi Province.

## Introduction

Owing to numerous health risks and tremendous increases in prevalence, overweight and obesity has gained recognition as a major public health concern in most middle- and high-income countries [1–3]. According to the latest estimates by the World Health Organization (WHO), 38.5 % of men and 39.6 % of women aged ≥18 years in the world were overweight, and 10.7 % of men and 15.2 % of women were obese in 2014 [4]. Rates of obesity in the world have more than doubled since 1980 [4]. China is a country currently suffering from serious threat of obesity and overweight, particularly in urban adults. The prevalence of overweight and obesity aged 18 and over increased substantially between 2002 and 2014, from 21.8 to 35.4 % in China [5, 6].

Excessive body weight is largely responsible for the dramatic increase in the prevalence of various chronic diseases, such as cardiovascular diseases, type 2 diabetes, musculoskeletal diseases, and some significant cancers which cause premature death and substantial disability, and can cause large life expectancy reductions in the population [7, 8]. Although the prevalence of overweight and obesity has reached alarming proportions in China, the problem of underweight remains unresolved in some remote and mountainous areas. China is a country facing the double burden of overweight and underweight. Many health dangers, such as immune system diseases, osteoporosis and bone loss, and various pregnancy complications, are associated with being underweight [9, 10]. Being underweight is a serious and under-recognized problem in China.

Apart from higher morbidity and mortality rates, abnormal body mass index (BMI) affects the health-related quality of life (HRQOL) in many ways. HRQOL is regarded as a multidimensional and comprehensive construct that measures the perceived impact of health or disease on the physical, mental and social functioning [11–14]. Previous studies revealed that obesity is associated with poorer physical functional HRQOL; however the impact of obesity on mental aspects of HRQOL is inconclusive [15–17]. The relationship between underweight and HRQOL is also mixed. Some studies have suggested that underweight is negatively associated with both the physical and mental aspects of HRQOL, however, other studies indicate that the underweight is associated with impairment of physical aspects rather than mental aspects of HRQOL, and a few have even reported that there is no association between underweight and HRQOL [18–20].

A few cross-sectional studies have assessed the relationship between BMI and HRQOL among adults in mainland China [21, 22]. These existing studies mostly focused on exploring the association between BMI categories and various dimensions of HRQOL. However, grouping BMI into pre-specified groups fails to reflect the nature of the relationship between BMI and HRQOL within BMI categories. This study aimed to investigate whether there is a nonlinear trend in the association between BMI and overall HRQOL using a semiparametric regression model, and to examine the relationship between BMI and five dimensions of HRQOL.

## Methods

### Data

The data used for analysis was derived from the fifth Health Service Survey of Shaanxi Province, which was part of China’s National Health Service Survey (NHSS), conducted in 2013. NHSS is a national representative survey commissioned by the Nation Health and Family Planning Commission of China. The detailed sampling and quality assurance measures have been described in a previously published paper [23]. In brief, a four-stage, stratified, random sampling scheme was used to ensure that samples are representative of the whole population of Shaanxi Province. In the first stage, 32 counties/districts were selected in Shaanxi Province. In the second stage, 160 townships were selected in sampled counties/districts. In the third stage, 320 villages/communities were selected in sampled townships. In the last stage, 20,700 households were identified. After verbal informed consent was obtained from interviewees, all members in each selected household were interviewed individually using a structured household questionnaire.

Among a total of 57,529 respondents, this study only focused on adult samples defined as those aged ≥18 years old (*n* = 47,151). Respondents whose HRQOL and/or BMI data were missing or not self-reported are further excluded in this analysis, leaving a study sample of 37,902 adult respondents.

### Ethical considerations

Approval for the fifth NHSS was given by the National Bureau of Statistics of China (license number 2013(65)). Approval for this cross-sectional study was obtained from the Ethics Committee of Xi’an Jiaotong University Health Science Center (approval number 2015–401).

### Variables

#### Body mass index

All respondents were asked to report their height and weight. BMI was calculated as the weight (in kilograms) divided by the square of the height (in meters) (kg/m^2^). In our study we explored the association between BMI and overall HRQOL scores by using continuous BMI, and assessed the association between BMI and various dimensions of HRQOL by grouping BMI into categories based on WHO International BMI classification criteria [24, 25]. Respondents were categorized into four groups: underweight (BMI < 18.5 kg/m^2^), normal weight (18.5 ≤ BMI < 25.0 kg/m^2^), overweight (25.0 ≤ BMI < 30.0 kg/m^2^), obese (BMI ≥ 30.0 kg/m^2^).

#### Health-related quality of life

The Chinese version EQ-5D-3 L, is preselected for the measurement of an individual’s HRQOL in the NHSS. The EQ-5D-3L consists of five dimensions: mobility (MO), self-care (SC), usual activity (UA), pain/discomfort (PD), and anxiety/depression (AD). Each dimension has 3 response levels, ranging from 1 (no health problems), 2 (moderate health problems), to 3 (extreme health problems). Overall, the EQ-5D-3L descriptive system defines a total of 243 unique health states. Although the EQ-5D-3L is the most widely used preference-based HRQOL measure in the world, the application of the EQ-5D-3L to measure HRQOL was restricted in China over the past few decades as the lack of Chinese population preference weights [26]. In this study, a recently developed Chinese-specific tariff (ranging from −0.149 for the worst health status, to 1 for the full health), based on the Chinese general population using the time trade-off method, has been adopted to score the EQ-5D-3L [27].

#### Other variables

Social-demographic characteristics considered in this study include sex, age, marital status (unmarried, married, divorced or widowed), education attainment (illiteracy, elementary, middle school, high school or university), residential areas (urban, rural), and economic status (grouped equally into the poorest, poorer, middle, richer, and the richest quintiles according to the per capita net household expenditure). Net household expenditure was calculated as total household expenditure in the last year minus household health expenditure [28]. In addition, physical activity (if respondents engaged in physical activities at least once a week in the last 6 months) and co-morbidities (i.e. a set of dummies indicating whether respondents had doctor diagnosed hypertension, diabetes, heart problems, musculoskeletal problems, respiratory disease or cancer) were also considered.

### Statistical analyses

Mean and standard deviation or proportions were used to describe the characteristics of the study samples, where appropriate.

To explore the potential non-linear relationship between continuous BMI and overall HRQOL in general adults, semiparametric regression models were adopted for analysis in our study [29]. Semiparametric regression model is a flexible model which allows us to mix parametric terms with nonparametric terms in the same model, and takes the following form:

$$ HRQOL=\alpha +f(BMI)+{\beta}_1^{\hbox{'}}{X}_1+{\beta}_2^{\hbox{'}}{X}_2+\varepsilon $$

where HRQOL denotes the EQ-5D-3L utility score, BMI is the BMI value, *f*(.) indicates that BMI variable is assumed to have a nonlinear effect on HRQOL and is fitted with nonparametric smoothers, X_1_ is a vector that contains socio-demographic characteristics and physical activity, X_2_ is a vector that refers to co-morbidities, β_1_, β_2_ are unknown coefficients to be estimated, α is the intercept and ε is the error term. In Model 1, only vector X_1_ was controlled, whilst in Model 2, vector X_2_ was further controlled. Men and women were analyzed separately.

Given the ceiling effects of the EQ-5D-3L instrument in the general population, we re-categorized the three response levels into two categories (having no problem and having any problems), for each of the EQ-5D-3L dimensions [30]. Binary logistic regression models were undertaken to assess the association between categorized BMI and the presence of problems in five dimensions of EQ-5D. The odds ratios (ORs) and corresponding 95 % confidence intervals (95 % CIs) were reported. Data analyses were carried out using the Stata software (version 10.0), and R statistical software with the mgcv package.

## Results

The characteristics of the study population are summarized in Table 1. A total of 37,902 adults, including 18,382 (48.50 %) men and 19,520 (51.50 %) women, participated in the study with the mean (standard deviation, SD) age of 50.01 (14.91) years and 48.71 (14.76) years in men and women, respectively. The mean (SD) BMI of respondents were 22.37 (2.82) kg/m^2^ and 21.98 (2.95) kg/m^2^ for men and women, respectively. According to the WHO International BMI cut-off, 7.20 %/10.92 % of men/women were underweight, 15.02 %/13.30 % of men/women were overweight, and 1.03 %/l.05 % of men/women were obese.

**Table 1:** Socio-demographic and clinical characteristics of the sample population

Characteristic	Men (*n* = 18,382)	Women (*n* = 19,520)
Age, years		
Mean (SD)	50.01 (14.91)	48.71 (14.76)
BMI, kg/m^2^		
Mean (SD)	22.37 (2.82)	21.98 (2.95)
EQ-5D utility score		
Mean (SD)	0.96 (0.11)	0.95 (0.12)
BMI WHO international categories		
Underweight, n(%)	1323 (7.20)	2132 (10.92)
Normal, n(%)	14,108 (76.75)	14,587 (74.73)
Overweight, n(%)	2761 (15.02)	2596 (13.30)
Obese, n(%)	190 (1.03)	205 (1.05)
Marital status		
Unmarried, n(%)	1654 (9.00)	864 (4.43)
Married, n(%)	15,560 (84.65)	16,782 (85.97)
Divorced or widowed, n(%)	1168 (6.35)	1874 (9.60)
Educational attainment		
Illiteracy, n(%)	1841 (10.02)	4214 (21.59)
Elementary, n(%)	4677 (25.44)	5210 (26.69)
Middle school, n(%)	7983 (43.43)	6964 (35.68)
High school, n(%)	2806 (15.26)	2140 (10.96)
University, n(%)	1075 (5.85)	992 (5.08)
Residential areas		
Urban, n(%)	5932 (32.27)	6839 (35.04)
Rural, n(%)	12,450 (67.73)	12,681 (64.96)
Physical activity		
No, n(%)	14,487 (79.04)	14,677 (75.52)
Yes, n(%)	3842 (20.96)	4757 (24.48)
Co-morbidities		
Hypertension, n(%)	2220 (12.08)	2903 (14.87)
Diabetes, n(%)	434 (2.36)	502 (2.57)
Heart problems, n(%)	359 (1.95)	500 (2.56)
Respiratory problems, n(%)	302 (1.64)	231 (1.18)
Musculoskeletal problems, n(%)	602 (3.27)	892 (4.57)
Cancer, n(%)	18 (0.10)	29 (0.15)

*BMI* body mass index

*WHO* World Health Organization

*SD* standard deviation

Table 2 shows the proportion of respondents reporting any problems in each of five dimensions of EQ-5D by weight status (BMI). As can be seen, among the four BMI groups underweight men reported the highest proportion of having problems in all five dimensions of EQ-5D. For women being obese reported the highest proportion of having problems in three EQ-5D dimensions (mobility, usual activity and pain/discomfort dimensions), whilst being underweight was identified for the left two dimensions (self-care and anxiety/depression). Among five EQ-5D dimensions, consistent results from unhealthy weight respondents suggest that pain/discomfort is the most severely impacted dimension, whilst self-care is the least impacted dimension.

**Table 2:** The proportion of respondents having any problems in each dimension of the EQ-5D-3L (%)

	Men				Women			
	Underweight	Normal weight	Overweight	Obese	Underweight	Normal weight	Overweight	Obese
MO	16.25	5.85	5.87	7.89	10.88	5.81	7.74	15.61
SC	8.62	3.13	2.57	2.11	6.19	3.13	4.20	5.85
UA	14.29	4.77	3.98	6.32	10.18	5.09	5.89	11.71
PD	27.59	12.50	10.65	14.21	21.95	14.36	19.07	28.29
AD	13.30	6.28	5.94	6.84	12.38	7.72	10.05	11.22

*MO* mobility, *SC* self-care, *UA* usual activity, *PD* pain/discomfort, *AD* anxiety/depression

Table 3 presents the estimated regression coefficients from semiparametric regression models for men and women, respectively. Among all the socio-demographic characteristics studied, age, marital status, educational attainment and economic status were all significantly associated with EQ-5D utility scores in both models. Being physically active was associated with better HRQOL. All co-morbidities were significantly associated with a decreased HRQOL (Model 2). Among them, respondents with cancer reported the largest decrement in EQ-5D utility score, with more than 0.176 and 0.157 point declines for men and women, respectively. Considering co-morbidities further improve the model fit that an adjusted R^2^ in Model 2 increased from 0.114 to 0.149 for men, and 0.141 to 0.188 for women.

**Table 3:** Estimated regression coefficients from the semiparametric models

	Men			Women	
	Estimate	Std. Error		Estimate	Std. Error
Model 1 (Adj R^2^ = 0.114)			Model 1 (Adj R^2^ = 0.141)		
Socio-demographic and physical activity			Socio-demographic and physical activity		
Intercept	1.010**	0.00521	Intercept	1.005**	0.00610
BMI	Estimated with 6.753 edf, see left plot of Figure 1		BMI	Estimated with 6.791 edf, see left plot of Figure 2	
Age	−0.0020**	0.00007	Age	−0.0021**	0.00007
Marital status			Marital status		
Unmarried(ref)			Unmarried(ref)		
Married	0.0249**	0.00308	Married	0.0192**	0.00436
Divorced or widowed	−0.0026	0.00453	Divorced or widowed	−0.0154**	0.00541
Educational attainment			Educational attainment		
Illiteracy(ref)			Illiteracy(ref)		
Elementary	0.0141**	0.00304	Elementary	0.0283**	0.00248
Middle school	0.0223**	0.00305	Middle school	0.0333**	0.00261
High school	0.0241**	0.00354	High school	0.0342**	0.00349
University	0.0248**	0.00471	University	0.0320**	0.00478
Residential areas			Residential areas		
Urban(ref)			Urban(ref)		
Rural	0.0001	0.00023	Rural	0.0002	0.00024
Economic status			Economic status		
Poorest(ref)			Poorest(ref)		
Poorer	0.0057*	0.00251	Poorer	0.0067*	0.00265
Middle	0.0119**	0.00254	Middle	0.0107**	0.00265
Richer	0.0093**	0.00261	Richer	0.0061*	0.00270
Richest	0.0126**	0.00270	Richest	0.0066*	0.00278
Physical activity			Physical activity		
No(ref)			No(ref)		
Yes	0.0039*	0.00216	Yes	0.0130**	0.00207
Model 2 (Adj R^2^ = 0.149)			Model 2 (Adj R^2^ = 0.188)		
Socio-demographic and physical activity			Socio-demographic and physical activity		
Intercept	0.9979**	0.00519	Intercept	0.9975**	0.00603
BMI	Estimated with 6.693 edf, see right plot of Figure 1		BMI	Estimated with 6.750 edf, see right plot of Figure 2	
Age	−0.0016**	0.00007	Age	−0.0017**	0.00007
Marital status			Marital status		
Unmarried(ref)			Unmarried(ref)		
Married	0.0227**	0.00305	Married	0.0144**	0.00430
Divorced or widowed	−0.0044	0.00448	Divorced or widowed	−0.0181**	0.00534
Educational attainment			Educational attainment		
Illiteracy(ref)			Illiteracy(ref)		
Elementary	0.0154**	0.00301	Elementary	0.0276**	0.00245
Middle school	0.0238**	0.00302	Middle school	0.0320**	0.00257
High school	0.0265**	0.00351	High school	0.0334**	0.00344
University	0.0268**	0.00466	University	0.0308**	0.00471
Residential areas			Residential areas		
Urban(ref)			Urban(ref)		
Rural	−0.0001	0.00023	Rural	0.0001	0.00023
Economic status			Economic status		
Poorest(ref)			Poorest(ref)		
Poorer	0.0063*	0.00248	Poorer	0.0067*	0.00260
Middle	0.0123**	0.00251	Middle	0.0104**	0.00261
Richer	0.0096**	0.00258	Richer	0.0058*	0.00266
Richest	0.0130**	0.00267	Richest	0.0061*	0.00274
Physical activity			Physical activity		
No(ref)			No(ref)		
Yes	0.0071**	0.00214	Yes	0.0147**	0.00205
Co-morbidities			Co-morbidities		
Hypertension	−0.0343**	0.00261	Hypertension	−0.0363**	0.00250
Diabetes	−0.0387**	0.00534	Diabetes	−0.0253**	0.00523
Heart problems	−0.0574**	0.00619	Heart problems	−0.0680**	0.00516
Respiratory disease	−0.0588**	0.00626	Respiratory disease	−0.0340**	0.00752
Musculoskeletal problems	−0.0821**	0.00441	Musculoskeletal problems	−0.0928**	0.00386
Cancer	−0.1767**	0.02517	Cancer	−0.1577**	0.02098

BMI Body mass index

*ref* reference category

*edf* effective degrees of freedom

*P < 0.05; ** *P* < 0.01

**Fig. 1:**
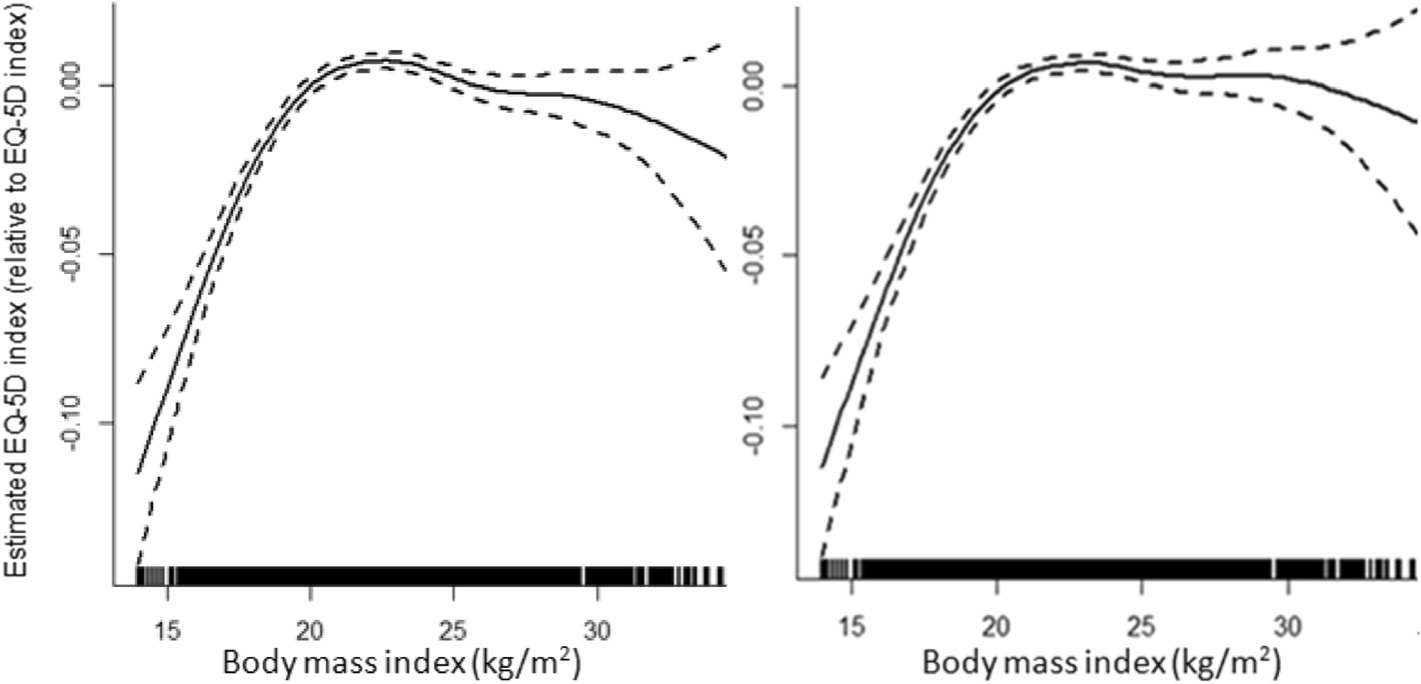
Nonparametric estimates for semiparametric regression model of EQ-5D for men. Estimates in the left plot are adjusted for age, marital status, educational attainment, residential area, economic status and physical activity. Estimates in the right plot further adjusted for co-morbidities. The dashed lines represent the 95 % confidence intervals. 10 participants with highest and lowest BMI values are not displayed because of estimation uncertainly for outliners

**Fig. 2:**
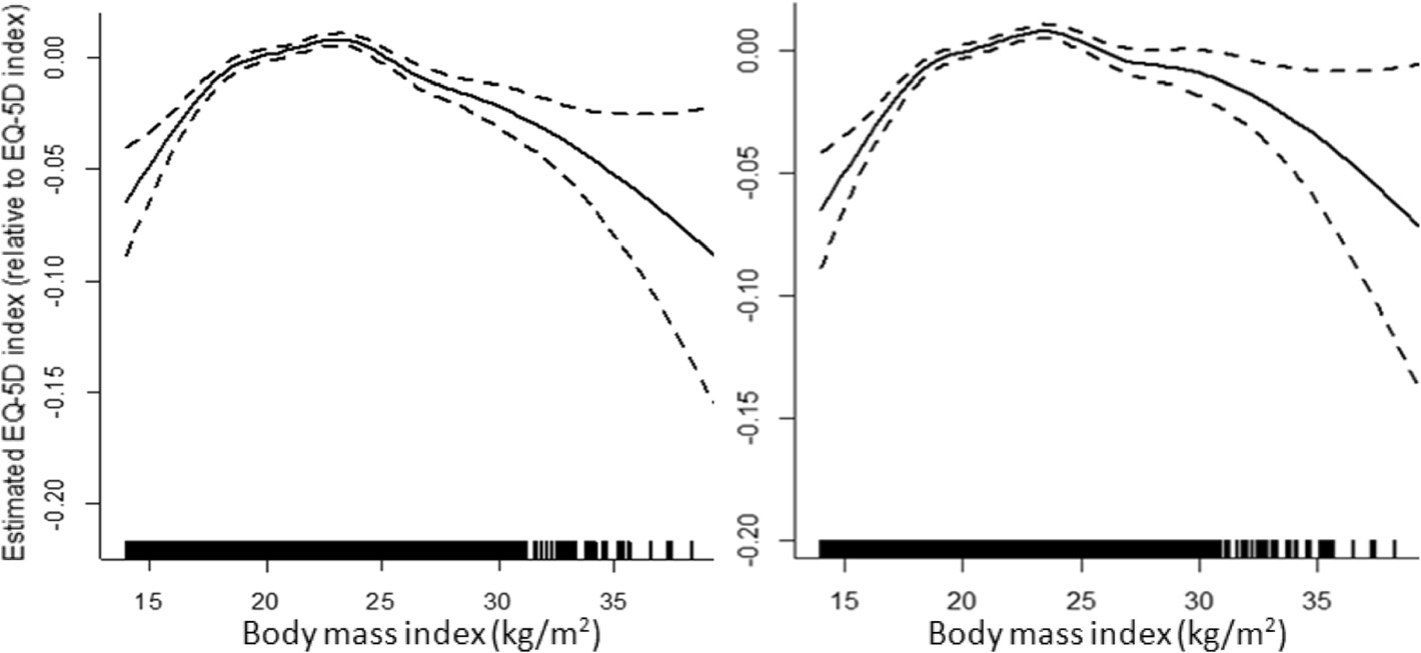
Nonparametric estimates for semiparametric regression model of EQ-5D for women. Estimates in the left plot are adjusted for age, marital status, educational attainment, residential area, economic status and physical activity. Estimates in the right plot further adjusted for co-morbidities. The dashed lines represent the 95 % confidence intervals. 7 participants with highest and lowest BMI values are not displayed because of estimation uncertainly for outliners

The nonlinear relationship between BMI and HRQOL for men and women are plotted in Figs. 1 and 2, respectively. The left plot in both figures refers to the model (Model 1) controlling for socio-demographic characteristics and physical activity; the right plot in both figures refers to the model (Model 2) which further controls for co-morbidities. With adjustment for socio-demographic and physical activity factors only, curves in the left plot showed that these are nonlinear relationships between BMI and HRQOL. When further controlled for co-morbidities, the nonlinear relationship between BMI and HRQOL remains, with optimal HRQOL achieved at a BMI of near 23 kg/m^2^ for men and 24 kg/m^2^ for women. Along with the increasing BMI values, EQ-5D utility scores decreased, especially in women. Between the BMI values 24 and 35, the mean EQ-5D utility scores decreased around 0.06 points for women in Model 1, whilst utility scores decreased around 0.04 points for the same BMI range when further controlled for co-morbidities. On the other hand, being underweight is also associated with a lower EQ-5D utility score. After controlling for co-morbidities, for BMI values 24 to 15, the mean EQ-5D utility scores decreased around 0.08 points for men, but 0.05 points for women.

Table 4 presents the results of binary logistic regression analyses of the five dimensions of the EQ-5D. Being underweight significantly increased the risk of suffering from problems in all five dimensions of HRQOL for both men and women, whilst the magnitudes of ORs were consistently larger for men than women. Overweight and obese men were significantly more likely to suffer from mobility problems, whilst overweight and obese women were significantly increased the risk of suffering from mobility and pain/discomfort problems.

**Table 4:** Association between BMI and HRQOL from logistic regression models [OR (95 % confidence interval)]^a^

	Men			Women		
	Underweight vs normal weight	Overweight vs normal weight	Obese vs normal weight	Underweight vs normal weight	Overweight vs normal weight	Obese vs normal weight
MO	2.47**(2.07–2.94)	1.24* (1.02–1.50)	1.76*(1.00–3.14)	1.37**(1.14–1.63)	1.30**(1.09–1.56)	2.03**(1.31–3.17)
SC	2.18**(1.74–2.75)	1.06(0.80–1.39)	0.85(0.30–2.37)	1.29**(1.03–1.61)	1.29(0.99–1.59)	1.14(0.59–2.20)
UA	2.51**(2.08–3.03)	1.03(0.82–1.29)	1.76(0.93–3.34)	1.45**(1.21–1.74)	1.09(0.89–1.33)	1.57 (0.96–2.57)
PD	2.17**(1.88–2.50)	0.92(0.79–1.06)	1.36(0.86–2.15)	1.39**(1.22–1.59)	1.23**(1.08–1.39)	1.49*(1.05–2.13)
AD	1.74**(1.45–2.09)	1.11(0.92–1.33)	1.19(0.65–2.16)	1.42**(1.22–1.66)	1.17 (0.99–1.35)	0.96(0.60–1.53)

BMI Body mass index

*MO* mobility, *SC* self-care, *UA* usual activity, *PD* pain/discomfort, *AD* anxiety/depression

*OR* odds ratio

*P < 0.05; **P < 0.01

^a^The relationship between BMI and HRQOL was analysed by logistic regression, adjusted for age, marital status, educational attainment, residential area, economic status, physical activity and co-morbidities

## Discussion

Using the world’s most widely used EQ-5D-3L instrument, this study estimated the relationship between continuous BMI and overall HRQOL scores among adults in Shaanxi Province by means of the semiparametric regression models. As expected, results revealed that the association between BMI and HRQOL is nonlinear (inverse U-shaped). More specifically, our study found that the EQ-5D-3L utility scores increased initially as BMI increased and achieved maximum at a BMI of around 23 kg/m^2^ for men and 24 kg/m^2^ for women, and then showed a decline trend with further increases of BMI. Before BMI reached optimal HRQOL, the EQ-5D utility scores were increasing faster among men than the women, whilst after the BMI value reached the optimal utility scores, women showed a faster decline in utility scores than men. The BMI value achieving optimal HRQOL in Shaanxi Province was lower than that value in the UK general population, which was estimated to be 24.5 in women and 27.5 in men [7].

Although this is the first study to explore whether there is nonlinear relationship between BMI and overall HRQOL scores in China using semiparametric regression models, earlier studies from other countries and specific populations provided evidence for nonlinear association between BMI and HRQOL. Soltoft et al. observed a nonlinear relationship between BMI and HRQOL in the general population of the UK [7]. Hunger et al. found the relationship between BMI and EQ-5D utilities were inverse U-shaped in individuals with type 2 diabetes [29]. After adjusting for covariates, Heo et al. observed J-shaped associations between BMI and HRQOL [31].

This study also explored whether the relationship between BMI and HRQOL could be mediated by co-morbidities. Our study found that regardless of whether adjusting for co-morbidities, the non-linear relationship is evident between HRQOL and BMI, although the magnitude of relationship decreased. This finding suggests unhealthy BMI is associated with decrements in HRQOL in nature, rather than this relationship just mediated through higher rates of co-morbidity. However, an exploratory study conducted in Singapore concluded that being obese no longer exhibited poor HRQOL after an adjustment for co-morbidities [18].

Previous studies revealed that being obese is associated with a lower physical HRQOL; however, the relationship between the obesity and mental domain of HRQOL are debated [3, 15, 21]. Consistent with many Asian studies, our study confirmed that, for both men and women, obesity negatively associated with physical rather than mental domain of HRQOL [18, 21]. Cultural and social difference could well explain why obese men were not associated with decrements in mental HRQOL. In Chinese traditional culture, excess weight is considered to be a symbol of happiness and wealthy for men [32]. The reason why obese women were not associated with decrements in mental HRQOL is that, although obese women may not satisfied with their fat body image, the mental health is not serious influenced by this weight-related distress. The relationship between BMI and physical domain of HRQOL varied between sexes. While obese women had higher risk of suffering from MO and PD problems, obese men just had higher risk of suffering from MO problem. The potential explanation is that women have higher pain sensitivity compared to men [33].

A lot of attention in the world is paid to dealing with the epidemic of overweight and obesity, however little attention is paid to assessing the relationship between underweight and HRQOL [34, 35]. Consistent with a previous study targeted at middle aged or older Chinese adults, our study found that underweight adults were significantly more likely to report having problems on all five EQ-5D-3L dimensions than those in the normal weight range [22]. In contrast to our study, another cross-sectional study, conducted in five major cities of China, reported that underweight respondents had similar physical and mental HRQOL compared to normal weight residents [21]. This discordant result may be due to sample selection limitations. Many studies in other Asian countries totally or partially supported our findings. One multiethnic study conducted in Singapore found that underweight reported the worst HRQOL compared to the pre-obese and obese [18]. Another study conducted in Japan found that being underweight was associated with impairment of physical aspects of HRQOL [19]. In view of the serious consequence of underweight (impairment of both physical and mental of HRQOL among adults in Shaanxi Province), interventions striving to tackle the prevalence of underweight should be put into action.

There are several strengths in this study. Firstly, the data we used were drawn from a large-scale cross-sectional survey conducted in Shaanxi Province. The large sample size of the Shaanxi’ NHSS gave our analysis excellent statistical power. Secondly, for the computing of EQ-5D utility scores, we used preference weights derived from the general Chinese population. Finally, an innovative statistical approach, semiparametric regression model, was used to explore the relationship between BMI and HRQOL without imposing any prior constraints of functional form.

There are some limitations that deserve consideration. Firstly, as the data we used was from a cross-sectional household survey, as such causal inferences about the relationship between BMI and HRQOL cannot be determined. Secondly, using the EQ-5D-3L instruments to assess the HRQOL has some limitations. As a generic preference-based instrument, EQ-5D-3L questionnaire may be less sensitive than an obesity-specific questionnaire to measure HRQOL. In addition, there is a notable ceiling effect for the EQ-5D-3L, particularly when it is used in the general population. However, a previous study has demonstrated that the EQ-5D-3L is not less sensitive to measuring HRQOL compared to the SF-6D, which is derived from the SF-36 [36]. Future studies could consider adopting the latest developed 5-level EQ-5D (EQ-5D-5L) to measure HRQOL. So far, the Chinese-specific tariff for EQ-5D-5L is unavailable. Thirdly, the weights and heights that were used to calculate BMI were self-reported. There is evidence suggesting that women tended to under-report their weight, whilst men tended to over-report their height [37]. Consequently, calculated BMI through self-reported data could be biased. However, self-reported height and weight were a major source of study on weight status in the large-scale household survey. In addition, many studies have also demonstrated that there is a strong correlation and high level of agreement between measured and self-reported BMI [38–40].

## Conclusion

The results from this study have demonstrated that there is a nonlinear relationship between BMI and HRQOL, regardless of whether controlling for co-morbidities. Consistent with most previous studies, our study found that obesity impaired the physical dimension rather than mental domain of EQ-5D. Underweight respondents had a high risk of suffering from both physical and mental problems. Interventions striving to tackle the prevalence of underweight should be put into action.

## References

[1] Silventoinen K, Sans S, Tolonen H, Monterde D, Kuulasmaa K, Kesteloot H, Tuomilehto J (2004). Trends in obesity and energy supply in the WHO MONICA Project. Int J Obesity.

[2] Ng M, Fleming T, Robinson M, Thomson B, Graetz N, Margono C, Mullany EC, Biryukov S, Abbafati C, Abera SF (2014). Global, regional, and national prevalence of overweight and obesity in children and adults during 1980–2013: a systematic analysis for the Global Burden of Disease Study 2013. Lancet.

[3] Huang IC, Frangakis C, Wu AW (2006). The relationship of excess body weight and health-related quality of life: evidence from a population study in Taiwan. Int J Obes (Lond).

[4] WHO: http://www.who.int/mediacentre/factsheets/fs311/en/(2015). Accessed 2 Feb 2015.

[5] WHO: Global health observatory data repository. http://apps.who.int/gho/data/node.main.A897C?lang=en. Accessed 10 Feb 2015

[6] Wu Y (2006). Overweight and obesity in China. BMJ.

[7] Soltoft F, Hammer M, Kragh N (2009). The association of body mass index and health-related quality of life in the general population: data from the 2003 Health Survey of England. Qual Life Res.

[8] Poirier P, Giles TD, Bray GA, Hong Y, Stern JS, Pi-Sunyer FX, Eckel RH (2006). Obesity and cardiovascular disease: pathophysiology, evaluation, and effect of weight loss. Arterioscler Thromb Vasc Biol.

[9] Flegal KM, Graubard BI, Williamson DF, Gail MH (2007). Cause-specific excess deaths associated with underweight, overweight, and obesity. JAMA.

[10] Flegal KM, Graubard BI, Williamson DF, Gail MH (2005). Excess deaths associated with underweight, overweight, and obesity. JAMA.

[11] Davalos ME, French MT (2011). This recession is wearing me out! Health-related quality of life and economic downturns. J Ment Health Policy Econ.

[12] Riazi A, Shakoor S, Dundas I, Eiser C, McKenzie SA (2010). Health-related quality of life in a clinical sample of obese children and adolescents. Health Qual Life Outcomes.

[13] Chen G, Ratcliffe J, Olds T, Magarey A, Jones M, Leslie E (2014). BMI, health behaviors, and quality of life in children and adolescents: a school-based study. Pediatrics.

[14] Rubin RR, Peyrot M (1999). Quality of life and diabetes. Diabetes Metab Res Rev.

[15] Larsson U, Karlsson J, Sullivan M (2002). Impact of overweight and obesity on health-related quality of life--a Swedish population study. Int J Obes Relat Metab Disord.

[16] Ul-Haq Z, Mackay DF, Fenwick E, Pell JP (2013). Meta-analysis of the association between body mass index and health-related quality of life among children and adolescents, assessed using the pediatric quality of life inventory index. J Pediatr.

[17] Renzaho A, Wooden M, Houng B (2010). Associations between body mass index and health-related quality of life among Australian adults. Qual Life Res.

[18] Wee HL, Cheung YB, Loke WC, Tan CB, Chow MH, Li SC, Fong KY, Feeny D, Machin D, Luo N (2008). The association of body mass index with health-related quality of life: an exploratory study in a multiethnic Asian population. Value Health.

[19] Takahashi Y, Sakai M, Tokuda Y, Takahashi O, Ohde S, Nakayama T, Fukuhara S, Fukui T, Shimbo T (2011). The relation between self-reported body weight and health-related quality of life: a cross-sectional study in Japan. J Public Health (Oxf).

[20] Jia H, Lubetkin EI (2005). The impact of obesity on health-related quality-of-life in the general adult US population. J Public Health (Oxf).

[21] Wang R, Wu MJ, Ma XQ, Zhao YF, Yan XY, Gao QB, He J (2012). Body mass index and health-related quality of life in adults: a population based study in five cities of China. Eur J Public Health.

[22] Zhu YB, Luo XX, Wang Q (2009). Study on the relationship between body mass index and health-related quality of life in middle-aged or older Chinese adults. Zhonghua Liu Xing Bing Xue Za Zhi.

[23] Xu Y, Gao J, Zhou Z, Xue Q, Yang J, Luo H, Li Y, Lai S, Chen G (2015). Measurement and explanation of socioeconomic inequality in catastrophic health care expenditure: evidence from the rural areas of Shaanxi Province. BMC Health Serv Res.

[24] WHO: BMI classification. http://apps.who.int/bmi/index.jsp?introPage=intro_3.html. Accessed 22 Jun 2015

[25] WHO (2001). Obesity: Preventing and Managing the Global Epidemic. Report of a WHO consultation on Obesity.

[26] Chen G, Khan MA, Iezzi A, Ratcliffe J, Richardson J (2015). Mapping between 6 Multiattribute Utility Instruments. Med Decis Making.

[27] Liu GG, Wu H, Li M, Gao C, Luo N (2014). Chinese time trade-off values for EQ-5D health states. Value Health.

[28] Chen G, Yan X (2012). Demand for voluntary basic medical insurance in urban China: panel evidence from the Urban Resident Basic Medical Insurance scheme. Health Policy Plan.

[29] Hunger M, Schunk M, Meisinger C, Peters A, Holle R (2012). Estimation of the relationship between body mass index and EQ-5D health utilities in individuals with type 2 diabetes: evidence from the population-based KORA studies. J Diabetes Complications.

[30] Tan Z, Liang Y, Liu S, Cao W, Tu H, Guo L, Xu Y (2013). Health-related quality of life as measured with EQ-5D among populations with and without specific chronic conditions: a population-based survey in Shaanxi Province. China Plos One.

[31] Heo M, Allison DB, Faith MS, Zhu S, Fontaine KR (2003). Obesity and quality of life: mediating effects of pain and comorbidities. Obes Res.

[32] Bin Li Z, Yin Ho S, Man Chan W, Sang Ho K, Pik Li M, Leung GM, Hing Lam T (2004). Obesity and depressive symptoms in Chinese elderly. Int J Geriatr Psych.

[33] Wiesenfeld-Hallin Z (2005). Sex differences in pain perception. Gender Med.

[34] Hongo M, Miwa H, Kusano M (2012). Symptoms and quality of life in underweight gastroesophageal reflux disease patients and therapeutic responses to proton pump inhibitors. J Gastroenterol Hepatol.

[35] Suastika K, Dwipayana P, Saraswati MR, Gotera W, Budhiarta AA, Sutanegara ND, Gunadi GN, Nadha KB, Wita W, Rina K (2012). Underweight is an important risk factor for coronary heart disease in the population of Ceningan Island. Bali Diab Vasc Dis Res.

[36] Sach TH, Barton GR, Doherty M, Muir KR, Jenkinson C, Avery AJ (2007). The relationship between body mass index and health-related quality of life: comparing the EQ-5D, EuroQol VAS and SF-6D. Int J Obes (Lond).

[37] Krul AJ, Daanen HA, Choi H (2011). Self-reported and measured weight, height and body mass index (BMI) in Italy, the Netherlands and North America. Eur J Public Health.

[38] Dekkers JC, van Wier MF, Hendriksen IJ, Twisk JW, van Mechelen W (2008). Accuracy of self-reported body weight, height and waist circumference in a Dutch overweight working population. BMC Med Res Methodol.

[39] Lucca A, Moura EC (2010). Validity and reliability of self-reported weight, height and body mass index from telephone interviews. Cad Saude Publica.

[40] Lv S, Su JX, Quan Y, Wu M (2012). Analysis on the knowing rates and accuracies of self-reported height, weight and waist circumstance data of adults. Jiangsu J Prev Med.

